# Bourdieu’s capitals matter: tracking longer working lives across Europe

**DOI:** 10.1007/s10433-026-00922-z

**Published:** 2026-04-29

**Authors:** Martin Lakomý, Filip Majetić, Muradiye Ateş

**Affiliations:** 1 Faculty of Business and Economics, Mendel University in Brno, Brno, Czechia; 2 Institute of Social Sciences Ivo Pilar, Zagreb, Croatia; 3 Department of Labor Economics and Industrial Relations, Ankara Hacı Bayram Veli University, Ankara, Türkiye; 4 Oxford Institute of Population Ageing, University of Oxford, Oxford, UK

**Keywords:** Older workers, Retirement transition, Capitals, Digitalisation, Inequalities, Welfare regime

## Abstract

Extending working lives is a major European policy goal, but the role of individual resources in retirement timing remains underexplored. To our knowledge, this study is the first to apply Bourdieu’s theory of capitals as a framework for understanding how resources/capitals affect retirement decisions over time. Using Waves 6–9 of panel data from Survey of Health, Ageing and Retirement in Europe, the study employs exploratory factor analysis to identify forms of capital and multilevel, time‑lagged logistic regression (observations nested within individuals) to explore how various forms of capital help older workers (N = 12,816) to remain in employment across three successive periods. The results show that human and health capital (dimensions of broader cultural capital) indeed help older adults to extend their working lives. The impact of other forms of capital (economic capital and dimensions of social capital) diminishes once relevant control variables are included, and the findings are robust across different time spans. Moreover, separate models by welfare regime capture substantial cross‑European variation, with some capitals predicting retirement only in certain welfare regimes. Overall, Bourdieu’s framework proves relevant in the current situation of population ageing and the digitalisation of economies. The paper suggests that policymakers should reflect on resource inequalities when developing measures to support longer working lives for those able to continue, while ensuring alternatives for those facing significant barriers.

## Introduction

This paper applies Bourdieu’s theory of capitals as a framework for a systematic exploration of the role of resources/capitals in retirement transitions in the context of rapid population ageing that reduces the proportion of the economically active population in Europe (Sobotka 2008; Timonen 2008). Policy largely prioritises prolonging working lives (Krekula and Vickerstaff 2017; Rašticová et al. 2020; Walker and Maltby 2012) but often neglects shortages of suitable jobs and persistent labour market inequalities in the European context. A key challenge for the ageing workforce is unequal access to essential competences, including digital skills, which reflects individual resources (Casas and Román 2024) rather than age alone. Another aspect of the topic is vast variation in digital skills among older workers, ranging from those without experience to the most proficient users (Helsper 2021; Lakomý 2023b). The convergence of ageing and digitalisation has increased the vulnerability and precarity of resource-limited older workers, resulting in adverse outcomes for them, their families, states, and employers struggling with labour shortages (Ní Léime et al. 2017). Workforce participation is contingent on access to skills and resources, which are shaped by institutional, occupational, and contextual factors rather than chronological age. Overall, theoretical and policy debates need clearer evidence on which resources enable longer, fulfilling work.

Bourdieu’s theory presents economic, cultural, and social capital as tools to build and reproduce social position (Bourdieu 1984, 1986). This framework explains how advantages in these forms of capital accumulate and reproduce over time, mediating the effects of work conditions, health, and other resources on retirement trajectories (Gilleard 2020). The forms of capital vary both across and within age groups, potentially influencing retirement trajectories and socioeconomic positions. Moreover, economic, cultural, and social capital can be expanded to include additional forms of resources that influence the situation of older workers: human capital (Becker 1964; Weller 2007; Gilleard and Higgs 2020), health capital (Grossman 2017; Schneider-Kamp 2021), and a less well-established concept of digital capital or skills (Helsper 2021; Lakomý 2023b).

All the forms of capital mentioned above can play an important role in retirement decisions, but this topic has not been fully explored in the previous research. Hence, this appears to be the first study to examine their interconnected influence, bringing the theoretical innovation of applying an expanded Bourdieusian framework to retirement transitions. This study demonstrates how considering health and digital resources within a multidimensional system shapes older workers’ scope for action during retirement transitions under conditions of increasing precarity in the digital age. It outlines a structure of capitals that connects classical and more recent components, using data from the longitudinal project Survey of Health, Ageing and Retirement in Europe (SHARE) and tests how all available forms of capital affect retirement decisions. SHARE provides nationally representative samples for populations aged 50 + . The paper examines the research question *‘How does economic, social, and cultural capital influence retirement decisions over time in Europe?’*, utilising the panel dimension of data to track actual retirement transitions across multiple waves. In addition, it examines how the role of different forms of capital varies across four welfare regimes (Esping-Andersen 1990; Fenger 2007; Ebbinghaus 2012). The main contribution is to identify a) the data-driven structure of concepts within Bourdieu’s framework, b) the effect of capitals over time, c) patterns of retirement transitions in the panel data, and d) the variation in these effects across the European context. These findings provide a theoretically grounded and empirically tested model of retirement behaviour for researchers and policymakers.

## Research background

Social capital, as defined by Bourdieu, is ‘the aggregate of the actual or potential resources which are linked… to membership in a group’ (Bourdieu 1986, p. 21) and accumulates in both work and non-work settings. In non-work settings, drawing on Putnam (2000), it is built through formal participation in advocacy groups, volunteering, and political activities as well as through informal socialising with family members, friends, acquaintances, and neighbours.

Previous studies on family-related social capital point to a strong effect of relationship status on early retirement. Family-related social capital acts as a ‘pull factor’ into retirement, as strong emotional ties and shared leisure aspirations reduce the relative value of employment and provide alternative sources of meaning outside paid work. Older Europeans who were partnered (Hochman and Lewin-Epstein 2013; Dingemans et al. 2017), or who had retired partners (Radl 2013), were more likely to retire early. Similar patterns have been observed in country-specific contexts, including among women in Norway (Dahl et al. 2003) and among both men and women in Czechia (Dudová and Pospíšilová 2022). Dutch interviews indicate that a desire to share leisure with a (especially older or non‑working) partner can pull individuals into early retirement (Reeuwijk et al. 2013; Sewdas et al. 2017). However, having children, likely reflecting the financial constraints of parenthood, has been associated with prolonged working lives across Europe (Dingemans et al. 2017).

Beyond close family ties, different aspects of non-work social connectedness are linked to distinct retirement patterns. In Germany, informal connectedness (socialising with friends, relatives, and neighbours) predicted earlier retirement, suggesting a substitution of leisure time for paid work, while formal connectedness (participation in voluntary and civic associations or local politics) predicted later retirement. From an active ageing and continuity perspective, combining later-life work with activities such as volunteering supports the maintenance of accustomed socio-professional roles (Lancee and Radl 2012).

The effect of social capital accumulated in the workplace contrasts with that derived from relationships with partners, friends, relatives, and neighbours. Across countries, at-work social connectedness emerged as a key motive for prolonging employment: interviewees emphasised continued contact with clients and colleagues in the Netherlands (Sewdas et al. 2017), belonging to a work team in Sweden (Hovbrandt et al. 2019), supportive relationships with colleagues and feelings of being appreciated in the UK (Stevens et al. 2022), and companionship, daily routine, and the prevention of loneliness in Ireland (Millman 2017).

Cultural capital exists in embodied, objectified, and institutionalised states (Bourdieu 1986). The embodied state refers to human cultivation, such as class-specific manners, tastes, or conversational styles; the objectified state comprises cultural goods (e.g. books); and the institutionalised state consists of formally recognised qualifications. Cultural capital includes several overlapping concepts, of which human, health, and digital capital are most relevant to this study. Human capital encompasses education, training, and cultivated skills. Health capital has been conceptualised as part of human capital (Goldin 2016), as embodied cultural capital (Gilleard and Higgs 2020), and as a stand-alone resource (Schneider-Kamp 2021). Digital capital—the accumulation of digital competences and access to technologies (Ragnedda 2018, p. 2367)—is understood either as a component of cultural capital (Morgan 2010; Calderón Gómez 2021) or as a stand-alone concept (Park 2017; Ragnedda 2018).

So far, cultural capital has been operationalised through education, digital skills, self-reported health, and occupational prestige. Higher educational attainment is consistently associated with later retirement across Europe (Dingemans et al. 2017; Axelrad and McNamara 2018; Pilipiec et al. 2022; Casas and Román 2023). While being both skilled and using a computer at work increased labour market participation among older Italian men (Biagi et al. 2013), changes in digital skills were not associated with changes in retirement intentions among European workers over time (Lakomý 2023b). Qualitative evidence from the Netherlands shows that it is not merely having knowledge and (digital) skills, but their active use at work, enabled by work design, that motivates continued employment; their underuse encourages earlier exit (Reeuwijk et al. 2013; Sewdas et al. 2017). Regarding occupational prestige, Western European ‘higher salariat’ and ‘petty bourgeoisie’ retire later than ‘skilled manual workers’, ‘higher grade blue-collar workers’, and ‘lower sales and service workers’ (Radl 2013).

Health capital, often conceptualised as embodied capacity influencing functional ability (e.g. intrinsic capacity literature; Cesari et al. 2018), is proxied by self-reported health measures in this study, consistent with conceptualisations of health capital in social theory (Schneider‑Kamp 2021). Across Europe, prolonged working lives are positively associated with self-reported well-being (Siegrist et al. 2007) and overall health (Hochman and Lewin-Epstein 2013; Dingemans et al. 2017; Axelrad and McNamara 2018; Lakomý 2023b). Country-specific evidence reinforces this pattern, with Dutch interviewees describing good health as a prerequisite for working beyond retirement age (Reeuwijk et al. 2013) and retirement decisions in the UK reflecting both the view that good health supports continued work and a ‘decline narrative’ in which delayed retirement is framed as a loss of time to enjoy remaining good health (Stevens et al. 2022).

Economic capital refers to financial resources, including monetary income and assets directly convertible into money, such as real estate, art, intellectual property rights, and business equity (Bourdieu 1986; Anheier et al. 1995; Cvetičanin et al. 2021). Evidence on its association with early retirement seems moderately conclusive. Across Europe, Hochman and Lewin-Epstein (2013) reported no association, whereas Aranki and Macchiarelli (2013) found that better-off individuals are more likely to retire early to pursue preferred lifestyles. Similarly, country-level evidence shows a positive influence of retirement income on early retirement intentions in Spain (Potočnik et al. 2009), while higher net-income older workers prefer earlier retirement in Norway (Dahl et al. 2003) and the Netherlands (Pilipiec et al. 2022). Dutch interviewees also emphasised financial circumstances, including access to early retirement schemes, employer or sector arrangements, and personal savings/assets, as crucial factors in retirement decision-making (Reeuwijk et al. 2013). Importantly, economic resources play a mediating role too, as they condition how other forms of capital can be mobilised by enabling their conversion. For example, it can mediate the conversion of social capital into spending more quality time with a partner, cultural capital into meaningful leisure or identity reconstruction or health capital into active lifestyles.

Finally, research identifies several individual- and country-level factors shaping retirement transitions. At the individual level, age is positively associated with retirement intentions (e.g. Harkonmäki et al. 2006), and women tend to retire earlier than men in both supranational (Dingemans et al. 2017; Casas and Román 2023) and national studies (Dudová and Pospíšilová 2022; Pilipiec et al. 2022). However, Radl (2013) shows that gender effects become less straightforward once occupational prestige and family circumstances are considered (e.g. close family ties and household wealth). Two work-related characteristics appear particularly relevant across Europe: self-employment (Axelrad and McNamara 2018; Casas and Román 2023; Lakomý 2023b) and longer job tenure (Hochman and Lewin-Epstein 2013) are both associated with prolonged working lives, reflecting economic necessity in the former case and maintenance of occupational prestige and/or accustomed socio-professional roles in the latter.

At the country level, higher unemployment rates, reflecting greater economic insecurity and vulnerability, have been linked to longer working lives (Axelrad and McNamara 2018; Lakomý and Zajíčková 2025). By contrast, technological change has been associated with earlier retirement due to higher job replacement (Casas and Román 2023), while the current level of digitalisation is associated with lower retirement intentions due to a more digitally proficient labour force (Lakomý and Zajíčková 2025). Pension system design also shapes retirement timing. Higher public pension expenditures reduce the likelihood of working beyond statutory retirement age, as they make retirement financially viable (Dingemans et al. 2017). Increases in the minimum retirement age encourage transitions into bridge jobs in Northern and Central Europe, while older workers are more likely to remain in full-time employment in Mediterranean countries, where fewer well-paid bridge jobs are usually available (Brunello and Langella 2013).

## Data and methods

### Data

The empirical part uses Waves 6–9 from SHARE. The SHARE database includes data from CAPI-based interviews with household members representing the population aged 50 + in participating countries, with 29 countries involved in at least one wave since 2004 (Börsch-Supan et al. 2013; Börsch-Supan 2022a, 2022b, 2022c, 2024). Response rates ranged from 40 to 62% across waves and types of response rates (Bergmann et al. 2019), with wave-to-wave retention around 80%, except for COVID-affected Wave 8 (Bergmann et al. 2022).

The analysis uses Wave 6 from 2015 as a baseline, as this is the only wave that includes the social networks module together with the IT module (and also the last wave measuring digital skills among a substantial proportion of the sample). Finally, the outcome variable indicating retirement transition is derived from Waves 7–9, with two-year gaps between the waves.

### Measurements

The dependent variable captures transition to retirement between the waves of data collection (0 = still working; 1 = entered retirement). The study excluded the unemployed (8.2% of the original sample), people registered as disabled (8.5%), and homemakers (11.5%) to focus on analytically relevant respondents and maintain conceptual clarity.

The capitals/resources were assessed using 17 indicators selected based on the conceptualisations presented in the “Research background” section. This set of variables included *household income* and *household net assets* (both variables in euros); *the ability to make ends meet* (from 1 = with great difficulty to 4 = easily); *workplace recognition*, *workplace support*, and *workplace autonomy* (all three from 1 = strongly disagree to 4 = strongly agree); *contact with family* and *contact with friends* (both from 1 = no contact to 7 = daily contact); *volunteering*, *participation in political organisations*, *reading*, and *visiting a sports or social club* (all from 1 = I did not to 5 = almost every day); *years of education* (0–25); *occupational prestige* (9 groups derived from ISCO-08 occupational classification, interpreted here as a ranking of occupations from lower to higher prestige, in line with Treiman’s (1977) concept of occupational prestige); *computer skills* (from 1 = never used to 6 = excellent); *chronic diseases* (0–9); and *mobility limitations* (0–6). The health indicators were reversed so that higher values indicate better health; the frequencies of activities were referenced to the last 12 months. Full labels of the indicators are provided in “Exploratory factor analysis—structure of capitals” section.

Based on the previously identified predictors of retirement transitions, the control variables included in the analysis were age, gender, partnership, number of children, employment type, and country. *Age*, ranging from 50 to 80, was accompanied by its quadratic term to reflect the nonlinear nature of the relationship. *Gender* was coded male = 0 and female = 1; not having a partner in the household = 0 vs *having a partner* = 1; *number of children* was top-coded at 9. Controlling for *employment type* (employee = 0; self-employed = 1) reflects the differences in the labour market positions and retirement transitions of these two groups. Finally, *the country of the respondent* was included in all models to account for differences in economic, cultural, and policy settings across individual European countries.

### Sample

Following Liu and De (2015), our final dataset was created through multiple imputation (MI) using the fully conditional specification (FCS) method. This decision stems from: a) the majority of the variables included in our study having missing values, b) the proportion of missing values ranging from 3.4 to 33.3% per variable, and c) the missing data pattern being ‘Missing at Random’ (MAR). Using FCS MI is also in line with the approach the SHARE organisation uses to create its ‘gv_imputations’ datasets.

The sample included 12,816 Europe-based workers aged 50 + , who reported being either employed (83% of the sample) or self-employed (17%) at the baseline Wave 6. The sensitivity analysis produced comparable findings in simplified models for a broader sample of workers, unemployed, disabled, and homemakers, but five variables are only available for workers, and thus, the final analysis includes those working in Wave 6. Then, the study applied the age restriction 50 + available in the data to retain the maximum amount of information and align with other studies of retirement transitions (Hochman and Lewin-Epstein 2013; Axelrad and McNamara 2018; Lakomý 2023b; Pilipiec et al. 2022), even if age definitions 55 + (Radl 2013) or 60 + (Dingemans 2017; Dudová 2022) can also be used for delineating older workers. The sample comprised respondents from 17 countries (see Table 3), ranging from 189 in Portugal to 1,579 in Estonia. The median age was 58 years (SD = 4.7), and 52% were women. Most respondents had completed secondary education (58%), followed by tertiary education (34%).

## Exploratory factor analysis—structure of capitals

The structure of capitals was derived using exploratory factor analysis (EFA). Because the 17-item variable set included both ordinal indicators (e.g. occupational prestige, Likert-type items) and continuous variables (e.g. years of education, number of chronic diseases), a mixed correlation matrix was computed using polychoric, polyserial, and Pearson’s correlations. The mixed correlation matrix was estimated using the *mixedCor()* function from the *psych* package in R (Revelle 2025). The optimal number of factors was evaluated using parallel analysis. EFA was conducted using minimum residual (minres) extraction and oblimin rotation, and items were retained if they loaded ≥ 0.50 on a single factor. Factor scores for the factors were computed using the ten Berge method and the scores were used in subsequent regression analyses. As these factor scores are inherently standardised, they were entered into the models as such, while other predictors were z-standardised to improve comparability across variables measured on different scales.

Although the analysis initially suggested extracting up to seven factors, inspection of the 7-, 6-, 5-, and 4-factor solutions demonstrated that only the four-factor structure was consistently well defined and interpretable; models with more factors produced empty factors or single-indicator factors. Therefore, a four-factor solution was retained. According to Table 1, the economic capital factor emerged as expected. Human capital, in line with its previous conceptualisations/operationalisations, encompassed individuals’ knowledge and skills (formal education and digital skills) as well as occupational prestige, while indicators of health capital formed an independent factor (Casas and Román 2023; Calderón Gómez 2021; Radl 2013; Schneider-Kamp 2021). In this factor structure, human and health capital can be understood as two dimensions of a broader cultural capital (Gilleard and Higgs 2020). Regarding social capital, unlike the indicators of at-work social capital that clustered together forming an independent factor labelled ‘workplace social capital’, the core indicators of non-work social capital (contact with family and contact with friends reflecting inner-circle social capital as well as volunteering and participation in political organisations reflecting wider community social capital) neither formed an independent factor nor loaded meaningfully onto any of the four retained factors, even under a more permissive loading criterion of ≥ 0.40. However, despite these variables failing to exhibit sufficient shared variance in any EFA solution, the relevance of social capital, both in Bourdieu’s theoretical framework and the previous empirical studies in the field (e.g. Dingemans et al. 2017; Lancee and Radl 2012), has prompted us to include them in subsequent regression analyses as individual observed indicators.

**Table 1:** Exploratory factor analysis: standardised loadings for the final 4-factor solution (≥ 0.50) *Source:*These calculations use data from SHARE, Wave 6.

Variable	Economic capital	Human capital	Health capital	Workplace social capital
Total household income	0.586			
Household ability to make ends meet	0.674			
Years of (formal) education		0.589		
Computer skills self-assessment		0.562		
Occupational prestige		0.593		
Number of chronic diseases			0.516	
Number of mobility limitations			0.659	
Perception of workplace support				0.723
Perception of workplace recognition				0.747

Variables not included in the table are contact with family; contact with friends; volunteering; participation in political and community organisations; perception of workplace autonomy; frequency of reading books/magazines; frequency of going to a sport or social club; and household net financial assets, as they were not part of any retained latent factor.

## Binary logistic regression—retirement transition

The EFA-yielded factors are utilised for estimating the effect of the capitals, measured in Wave 6, on retirement transitions in Waves 7–9 within the framework of time-lagged multilevel logistic regression, with observations of the outcome across three waves nested in individuals (“Pooled models for three subsequent waves” section). An additional sensitivity analysis shows that the coefficients are stable regardless of which wave is used to measure the outcome (working vs. retiring), allowing all outcome waves to be analysed jointly within the multilevel models. The initial model estimates the effect of EFA-yielded capitals with wave and country as the sole control variables, Model 2 includes the observed individual indicators of social capital, and Model 3 incorporates the remaining control variables. The lagged measurement partially mitigates the risks associated with endogeneity, including potential reverse causality, simultaneity bias, and omitted-variable bias (Hill et al. 2021; Wang and Bellemare 2019).

The second step of the regression analysis (“Differences between European welfare regimes” section) estimates the full model separately for groups of European countries defined by a welfare regime typology (Esping-Andersen 1990; Fenger 2007; Ebbinghaus 2012). This typology is based on differences in European welfare systems regarding their degree of de‑commodification, stratification, and the balance between state, market, and family responsibility for social protection, while also reflecting similarities in economic, cultural, and policy contexts. The welfare-regime grouping strategy has been used in prior studies (Brunello and Langella 2013; Sirovátka et al. 2019; Lakomý 2023a) to capture European diversity through relatively homogeneous clusters rather than to estimate the causal effects of welfare regimes directly. The analytical part identifies Denmark and Sweden as social-democratic countries; Austria, Belgium, France, Germany, Luxembourg, and Switzerland as continental countries; Greece, Italy, Portugal, and Spain as Mediterranean countries; and Croatia, Czechia, Estonia, Poland, and Slovenia as post-communist countries. Limited sample sizes do not allow for country-specific models, and the small number of countries does not permit multilevel modelling with countries as an additional level of measurement (Bryan and Jenkins 2016), so the study presents welfare regime differences while acknowledging country variation within these groups. All models are estimated using the *melogit* command in Stata 16.

### Pooled models for three subsequent waves

Table 2 shows three binary multilevel logistic regression models. The table displays odds ratios as more readily interpretable coefficients, while the factors are standardised (mean = 0, SD = 1). In Model 1, retirement has the strongest association with human capital—each one-standard-deviation increase in this factor decreases the odds of retiring by 48%—and health capital, decreasing the odds by 35% (both effects are stable across waves). In contrast, economic capital and workplace social capital increase the odds of retirement transitions. Overall, some forms of capital are connected to retiring earlier and some to retiring later when individual characteristics are not included in the model. Model 2 shows similar coefficients for the capitals, except for economic capital (which became much weaker with *p* = 0.052). From the added variables, stronger contact with family increases the odds of retiring by 126%, while volunteering decreases the odds by 12%. Nevertheless, these associations may be biased by some omitted characteristics, which are addressed in the last model.

**Table 2:** Odds ratios of binary logistic regression estimating transition to retirement across Waves 7–9 (observations), nested in individuals *Source:*These calculations use data from SHARE, Waves 6–9

	Model 1	Model 2	Model 3
Economic capital	1.317***	1.156	0.923
Human capital	0.519***	0.641***	0.646***
Health capital	0.647***	0.605***	0.889**
Workplace social capital	1.374***	1.299***	0.945
Contact with family		2.264***	0.983
Contact with friends		1.041	0.975
Participation in volunteering		0.884*	0.982
Participation in political and community organisations		1.013	1.023
Age			142.920***
Age squared			0.966***
Gender (woman)			1.394***
Partner in household (yes)			1.427***
Number of children			0.955
Self-employed (yes)			0.218***
*Wave of the outcome (Wave 7 is the reference)*			
Wave 8	22.318***	22.114***	17.164***
Wave 9	173.385***	174.226***	147.684***
Country (16 dummies)	Not shown	Not shown	Not shown
Nagelkerke R^2^	0.31	0.32	0.55
N—observations	27,514	27,514	27,514
N—respondents	12,816	12,816	12,816

*Significance levels:* *p < 0.05, **p < 0.01, ***p < 0.001.

Model 3 illustrates that coefficients change once control variables are added. Additional testing showed that controlling for age diminished some former associations. In particular, economic capital, workplace social capital, and the observed indicators of social capital are no longer significant. Interestingly, economic capital is the only factor that does not affect retirement uniformly; an additional analysis shows that income is connected to lower odds of retirement, while a better ability to make ends meet is associated with higher odds. The two remaining factors connected to lower odds of retirement in the first set of models also hold in the second set. Specifically, an increase in human capital by one standard deviation decreases the odds of retiring by 35%, while an increase in health capital decreases the odds by 11%.

### Differences between European welfare regimes

The final section of the analysis explores differences in the roles of the capitals across welfare regimes regarding the odds of retirement. Each welfare regime contains several countries listed under Table 3, with models controlling for country dummies and reflecting grouping‑level heterogeneity. These separate models show that forms of capital predict retirement transition differently across Europe, with the most notable role of the capitals in *continental countries*. In this group of countries, the odds of retirement are about 28% lower for workers with (one standard deviation) higher human capital, as well as higher economic capital and to some extent higher health capital (*p* = 0.052). In contrast, health capital and social capital (contacts with family and volunteering) appear to outweigh economic capital and human capital in *social-democratic countries*. Finally, the retirement transition in *Mediterranean countries* and *post-communist countries* is predicted by lower human capital (this factor reduces the odds of retirement by 20% in *Mediterranean countries* and by 64% in *post-communist countries*) as the only important factor in these groups of countries. As for the social capital indicators, only participation in political organisations increases the odds of retiring in *post-communist countries* (which is in contrast with the opposite effect of political organisations in *social-democratic countries* with the borderline *p* = 0.059). Additional analysis tested interactions of welfare regimes with all capitals, and the interaction tests indicate that all differences in odds ratios larger than 0.3 in Table 3 are statistically significant. Overall, the role of capitals in the retirement transition varies profoundly across Europe, while the sample sizes do not allow for country-specific models.

**Table 3:** Odds ratios of binary multilevel logistic regression estimating transition to retirement across European welfare regimes *Source:* These calculations use data from SHARE, Waves 6–9

	Social-democratic countries	Continental countries	Mediterranean countries	Post-communist countries
Economic capital	0.978	0.810**	1.094	1.389
Human capital	0.831	0.718**	0.798*	0.355***
Health capital	0.759*	0.868	0.962	0.943
Workplace social capital	0.918	0.904	0.958	0.963
Contact with family	0.739*	0.994	0.993	0.997
Contact with friends	1.039	0.986	0.980	0.976
Participation in volunteering	0.740*	1.012	0.977	1.149
Participation in political and community organisations	0.794	0.997	1.043	1.227*
Age	1465.499***	639.109***	9.395***	65.900***
Age squared	0.949***	0.955***	0.987***	0.971***
Gender (woman)	2.142***	0.973	0.838	2.380***
Partner in household (yes)	2.129*	1.935***	1.575*	0.983
Number of children	0.942	0.923	0.882	1.042
Self-employed (yes)	0.084***	0.070***	0.359***	0.790
*Wave of the outcome (Wave 7 is the reference)*				
Wave 8	18.257***	28.260***	11.572***	12.874***
Wave 9	160.370***	321.571***	93.993***	90.980***
Country (dummies)	Not shown	Not shown	Not shown	Not shown
Nagelkerke R^2^	0.58	0.59	0.51	0.53
N—observations	4,858	9,967	5,100	7,589
N—respondents	2,165	4,501	2,674	3,476

Social-democratic countries are represented by Denmark and Sweden; continental countries by Austria, Belgium, France, Germany, Luxembourg, and Switzerland; Mediterranean countries by Greece, Italy, Portugal, and Spain; and post-communist countries by Croatia, Czechia, Estonia, Poland, and Slovenia

*Significance levels: p* * < 0.05, **p < 0.01, ***p < 0.001.

## Discussion and conclusions

Drawing on Bourdieu’s theory of capitals, this paper examines how older workers’ economic, cultural, and social resources shape retirement transitions over time using longitudinal SHARE data. To operationalise these resources, exploratory factor analysis derives forms of capital, identifying economic capital, human capital, health capital, and workplace social capital, with human and health capital together representing broader cultural capital. As indicators of non-work social capital did not form a coherent factor, inner-circle and wider community social capital are captured using four observed indicators. Subsequent analyses show that selected forms of capital systematically shape retirement transitions and vary across European welfare regimes, reflecting the idea that the value and convertibility of capitals depend on institutional contexts.

Economic capital affects retirement transitions, but effects differ by its components. In particular, the more objective part of economic capital *(income) reduces the odds of retirement, while its more subjective component (perceived ability to make ends meet) increases it*. These results are in line with the mixed findings of previous studies (Aranki and Macchiarelli 2013; Dahl et al. 2003; Hochman and Lewin-Epstein 2013; Pilipiec et al. 2022). The section focusing on welfare regimes indicates that the role of economic capital substantially differs across Europe; it reduces the odds of retirement in *continental countries* and increases the odds in post-communist countries, where its convertibility into retirement options is shaped by lower pension generosity.

Cultural capital stands out as a central resource, comprising health capital and human capital as two particularly relevant yet often overlooked components. First, *health capital emerged as the most significant predictor of retirement transition*, with better health capital being associated with delayed retirement in line with existing research (Axelrad and McNamara 2018; Dingemans et al. 2017; Hochman and Lewin-Epstein 2013; Lakomý 2023b). Second, human capital, reflecting occupational prestige, formal education, and digital skills, substantially predicts prolonged labour force participation. Our findings support the argument that *digital skills are an important resource for older workers* (Lakomý 2023b), which are becoming increasingly relevant given a) rapid technological development, b) labour shortages, and c) COVID-related reorganisation (Schaupp 2023). While health capital helps extend working life only in *social-democratic countries*, human capital strongly delays retirement in *post-communist countries* but has no effect in *social-democratic countries*. This contrast suggests that the convertibility of cultural capital differs across welfare‑state contexts, with universalistic systems making health‑based work capacity more salient and post‑communist systems placing greater weight on mobilising skills. If this is the case, then the *implementation of AI (*Casas and Román 2024*) could make human capital much more important for older workers,* and the EU-level bodies should coordinate the development of lifelong learning across countries.

Workplace social capital appears to the odds of retirement, but this effect diminishes when individual characteristics are included. This is in line with qualitative findings that continued contact with clients and colleagues in the Netherlands was stated as a key motive for prolonging employment (Sewdas et al. 2017). The overall effect of inner circle (contact with family and friends) and wider community social capital indicators (volunteering and political organisations) is negligible, but the welfare-regime-specific analysis identified a consistent beneficial role of these resources in the *social-democratic countries*. These findings may reflect that intergenerational relationships are understood less as obligations and more as opportunities in Nordic countries (Marckmann 2017), implying that value of social ties depends on social norms that define which forms of engagement are recognised and potentially convertible to other resources. Overall, these findings demonstrate the need to consider how different forms of social capital shape retirement transitions, emphasising the importance of incorporating social context into retirement policy frameworks.

This study has several limitations, related to the use of existing survey data. First, the indicators of capitals are constrained by items in the data; e.g., digital skills could be measured in a more elaborate way. Some questions on social capital and human capital were asked only once, which limits some strategies for panel data analysis. Second, the panel sample faces attrition, and the sample size of the working/retired part does not allow country-specific analysis. Third, the lagged measurement reduces endogeneity issues but cannot provide robust causal evidence. Fourth, 17 European countries vary in their pension systems and labour market contexts, and thus, the welfare regime differences reflect multiple contextual factors. While the presented analysis cannot account for this variation—and between-country multilevel modelling is not possible because of a small number of countries (Bryan and Jenkins 2016)—controlling for country, wave/year, age, and employee/self-employed status serves as an indirect proxy for retirement eligibility. Furthermore, the analysis aims for context sensitivity by splitting the sample by four welfare regimes; this step demonstrated that some predictors work consistently, while others depend on the macro context.

This study shows how different forms of capital shape retirement transitions in Europe across three subsequent waves and may inspire similar research elsewhere. It also demonstrates that Bourdieu’s theory of capitals is a useful framework for studying retirement transitions, but that it requires some adaptation in this field. A first theoretical insight concerns economic capital. Although central to Bourdieu’s original formulation (Bourdieu 1986), economic capital does not show a consistent direct effect on retirement transitions, indicating that its main role lies in enabling the mobilisation and conversion of other forms of capital. A second insight relates to social capital: a relatively standard set of social-capital indicators does not form one or more coherent factors, suggesting greater conceptual and empirical fragmentation of this form of capital in the context of retirement. In contrast, cultural capital can be disentangled into health and human capital (the latter having a digital component), which both prolong working lives. Finally, the overall pattern varies substantially across European welfare regimes, supporting the argument that effects of capitals are conditioned by welfare regimes or another macro-structure. In sum, this paper provides *a context-sensitive evaluation of how forms of capital shape retirement transitions over time*, which represents its key contribution to both theory and practical understanding of retirement behaviour.

## Data Availability

The dataset is accessible to the public after registration. The authors will share the syntax upon request.
